# Phylogenetic lineages and antimicrobial resistance determinants of clinical *Klebsiella oxytoca* spanning local to global scales

**DOI:** 10.1128/spectrum.00549-23

**Published:** 2023-09-07

**Authors:** Odion O. Ikhimiukor, Stephanie S. R. Souza, Ifeoluwa J. Akintayo, Michael M. Marcovici, Adrienne Workman, Isabella W. Martin, Cheryl P. Andam

**Affiliations:** 1 Department of Biological Sciences, University at Albany, State University of New York, Albany, New York, USA; 2 Institute for Infection Prevention and Hospital Epidemiology, Medical Centre, University of Freiburg, Freiburg, Germany; 3 Department of Pathology and Laboratory Medicine, Dartmouth-Hitchcock Medical Center, Lebanon, New Hampshire, USA; University of Sydney, Darlington, New South Wales, Australia

**Keywords:** *Klebsiella oxytoca*, bloodstream, genome, antimicrobial resistance, *bla*_OXY-2_

## Abstract

*Klebsiella oxytoca* is an opportunistic pathogen causing serious nosocomial infections. Knowledge about the population structure and diversity of healthcare-associated *K. oxytoca* from a genomic standpoint remains limited. Here, we characterized the phylogenetic relationships and genomic characteristics of 20 *K*. *oxytoca sensu stricto* isolates recovered from bloodstream infections at the Dartmouth-Hitchcock Medical Center, New Hampshire, USA from 2017 to 2021. Results revealed a diverse population consisting of 15 sequence types (STs) that together harbored 10 variants of the intrinsic beta-lactamase gene *bla*_OXY-2_, conferring resistance to penicillins. Similar sets of antimicrobial resistance (AMR) determinants reside in multiple distinct lineages, with no one lineage dominating the local population. To place the New Hampshire *K. oxytoca* in a broader context, we compared them to 304 publicly available genomes of clinical isolates from 18 countries. This global clinical *K. oxytoca sensu stricto* population is represented by over 65 STs that together harbored resistance genes against 14 antimicrobial classes, including eight *bla*_OXY-2_ variants. Three dominant STs in the global population (ST2, ST176, ST199) circulate across multiple countries and were also present in the New Hampshire population. The global *K. oxytoca* population is genetically diverse, but there is evidence for broad dissemination of a few lineages carrying distinct set of AMR determinants. Our findings reveal the clinical diversity of *K. oxytoca sensu stricto* and its importance in surveillance efforts aimed at monitoring the evolution of this drug-resistant nosocomial pathogen.

**IMPORTANCE:**

The opportunistic pathogen *Klebsiella oxytoca* has been increasingly implicated in patient morbidity and mortality worldwide, including several outbreaks in healthcare settings. The emergence and spread of antimicrobial resistant strains exacerbate the disease burden caused by this species. Our study showed that clinical *K. oxytoca sensu stricto* is phylogenetically diverse, harboring various antimicrobial resistance determinants and *bla*_OXY-2_ variants. Understanding the genomic and population structure of *K. oxytoca* is important for international initiatives and local epidemiological efforts for surveillance and control of drug-resistant *K. oxytoca*.

## INTRODUCTION

*Klebsiella oxytoca* is a Gram-negative, rod-shaped, facultative anaerobic bacterium belonging to the family *Enterobacteriaceae*. This ecologically versatile bacterium has been isolated in soil, water, human and animal sources (1, 2). In humans, it is commonly found as a commensal in the gut, and has also been isolated from the skin, urinary tract, oral cavity, and respiratory tract (3 – 5). It is also an opportunistic pathogen that has been increasingly reported in nosocomial infections (6). *K. oxytoca* has been implicated in diarrhea (7), ventriculitis (8), keratitis (9), urinary tract infections (3), antibiotic-associated hemorrhagic colitis (10), necrotizing entercolitis (11), bacteremia (12), meningitis (13), and pyogenic liver abscess (14). While its prevalence in clinical settings is less than the more frequently studied *Klebsiella pneumoniae*, *K. oxytoca* is well adapted to healthcare environments (2, 15). Outbreaks of *K. oxytoca* have been reported in different hospital wards, such as neonatal and pediatric intensive care units, oncology, and renal transplantation (16 – 19).

The increasing resistance of *K. oxytoca* against many commonly used antimicrobials presents an alarming health threat and complicates options for therapy and infection control (6, 20). Mechanisms of antimicrobial resistance in *K. oxytoca* are remarkably varied. *K. oxytoca* carries intrinsic antimicrobial resistance (AMR) genes. The most notable is the *bla*_OXY_ encoding the class A beta-lactamase OXY, which is constitutively expressed at low levels to confer resistance to aminopenicillins (e.g., ampicillin, amoxicillin), carboxypenicillins (e.g., carbenicillin, ticarcillin), and other penicillins (21, 22). Although *bla*_OXY_ is typically found in the chromosome, a plasmid-encoded OXY beta-lactamase has been previously reported in *K. oxytoca* isolates from a hematology unit (22). Another intrinsic AMR determinant is the efflux pump-encoding *oqxA-oqxB* that mediates low-level resistance to quinolones (5). AMR genes carried by mobile genetic elements and disseminated through horizontal gene transfer are also pervasive in *K. oxytoca* (15). Horizontal gene transfer facilitates acquisition of additional AMR genes by *K. oxytoca* from other species of *Klebsiella* and Enterobacterales (23, 24). Reduced susceptibility to quinolones may be conferred through non-synonymous point mutations in chromosomal genes such as DNA topoisomerase II subunit A (*gyrA*) and DNA topoisomerase IV subunit A (*parC*) (25, 26), while colistin resistance is associated with changes in *mgrB,* the negative regulator of the PhoP-PhoQ two-component system (27). Reported outbreaks of *K. oxytoca* in healthcare settings often involved multidrug-resistant strains that carry extended-spectrum beta-lactamases and carbapenemases (17 – 19, 28).

However, knowledge about the genetic diversity of *K. oxytoca*, including the identity of circulating lineages and AMR determinants, remains inadequate. This is mainly because *K. oxytoca* is often overshadowed by its more notorious relative *K. pneumoniae* in hospital settings, its long history of complicated taxonomy, and challenges in phenotypically distinguishing it from eight other closely related species of the *K. oxytoca* species complex (5, 29). Here, we aim to characterize the phylogenetic relationships and genomic features of clinical *K. oxytoca sensu stricto* at two geographical scales. First, we sequenced the genomes of 20 *K*. *oxytoca* isolates recovered from patients with bloodstream infections at Dartmouth-Hitchcock Medical Center (DHMC), New Hampshire, USA. Next, we combined them with the global *K. oxytoca sensu stricto* population from clinical sources (*n* = 304 genomes). Results showed that clinical *K. oxytoca sensu stricto* is phylogenetically diverse, harboring multiple AMR genes including several *bla*_OXY-2_ variants. However, we found that clinical *K. oxytoca sensu stricto* at the hospital level can be attributed to multiple lineages carrying similar AMR determinants, but its success at the global geographical scale may have been influenced by the presence of distinct sets of AMR determinants carried by multiple lineages. Our findings have important implications for international initiatives and local epidemiological efforts for surveillance and control of drug-resistant *K. oxytoca*.

## MATERIALS AND METHODS

### Bacterial collection, identification, and antimicrobial susceptibility testing

We obtained a total of 232 *Klebsiella* isolates from bloodstream infections in unique pediatric and adult patients. Isolates were grown from clinical blood culture specimens submitted to the Department of Pathology and Laboratory Medicine at DHMC, New Hampshire, USA from February 2017 to November 2021. Initial species identification was carried out using either the FilmArray Blood Culture Identification (BCID) panel (February 2017 through May 2021) or the FilmArray Blood Culture Identification 2 (BCID2) panel (May 2021 through November 2021). The BCID/BCID2 panel is a sample-to-answer multiplexed PCR assay for rapid identification of the causative pathogen from positive blood cultures (30). Of the isolates studied, 49 isolates were identified in the clinical laboratory as *K. oxytoca* using this assay.

The first significant blood culture isolate from each patient is routinely archived in case of future need for patient care, epidemiologic, public health, or laboratory quality studies. Upon subculture, isolates were assigned a study number and all patient identifiers were removed with only the date of collection and results of clinical antimicrobial susceptibility testing linked to the study number. Antimicrobial susceptibility testing was performed on the MicroScan Walkaway 96 Plus automated instrument (Beckman-Couter, La Brea, CA, USA) using the NUC62 Panel from January 2017 through May 2019, then switching to the Neg MIC Panel 46 from June 2019 onwards. The antimicrobial agents tested included seven antimicrobial classes (Table S1): aminoglycosides (amikacin, gentamicin); antifolate (sulfamethoxazole/trimethoprim); beta-lactams (cefoxitin), carbapenems (ertapenem, meropenem); cephalosporins (cefazolin, cefepime, cefotaxime, cefotetan, ceftazidime, ceftriaxone, cefuroxime); fluoroquinolones (ciprofloxacin, levofloxacin); monobactam (aztreonam); penicillins (ampicillin, ampicillin-sulbactam, amoxicillin-clavulanic acid, piperacillin/tazobactam). All isolates were stored in DMSO solution in −80°C.

### DNA extraction, library preparation, and whole genome sequencing

Isolates were subcultured from DMSO stocks onto commercially prepared tryptic soy agar with 10% sheep red blood cells (Remel, Lenexa, KS, USA) and in brain heart infusion broth (BD Difco, Franklin Lakes, NJ, USA) at 37°C for 24 h. DNA was extracted and purified from liquid cultures using the Zymo Research QuickDNA Fungal/Bacterial Miniprep Kit (Irvine, CA, USA) following manufacturer’s protocol. We used Qubit fluorometer (Invitrogen, Grand Island, NY, USA) to measure DNA concentration. DNA libraries of each sample were prepared using the Illumina DNA Prep Kit and IDT 10 bp UDI indices in accordance with the manufacturer’s instructions.

DNA samples were sequenced as multiplexed libraries on the Illumina NextSeq 2000 platform operated per the manufacturer’s instructions. Sequencing resulted in 151-nt long paired end reads. Sequencing was carried out at the SeqCenter (Pittsburg, PA, USA). Illumina bcl-convert (v.3.9.3) was used for demultiplexing, quality control, and adapter trimming of reads.

### *De novo* genome assembly, sequence quality check, and annotation

Paired end read sequences were assembled into contigs using shovill v1.1.0 (https://github.com/tseemann/shovill). Trimming of adapter sequences was enabled using the trim option. Shovill includes methods for subsampling read depth down to 150X, trimming adapters, correcting sequencing errors, and assembling using SPAdes v.3.14.1 (31). We used QUAST v.5.0.2 (32) and CheckM v.1.1.3 (33) to assess quality of assembled genomes. We calculated the genome completeness (mean = 99.87%; range: 98.56–99.99%) and genome contamination (mean = 0.49%; range = 0.31–0.97%), which were all within the genome quality standards recommended by CheckM (Table S2; Fig. S1). We also excluded assemblies with >200 contigs and an N50 <40,000 bp. The number of contigs range from 29 to 98 (median: 42.5) and N50 of 135,461–565,207 bp (median: 358,312 bp) (Table S2; Fig. S1). The assembled draft genomes were annotated using Prokka v.1.14.6 (34). The annotated genomes were used as input to characterize the pan-genome, i.e., the totality of genes of all strains in our data set, using Roary v.3.13.0 (35). Nucleotide sequences were aligned using MAFFT v.7.471 (36).

### Species confirmation

Members of the *K. oxytoca* species complex, which consists of nine species (5), are difficult to discriminate both phenotypically and by standard laboratory assays (5). Hence, they are all usually reported as *K. oxytoca* by hospitals and clinical laboratories. To validate species identity, we used Kleborate v.2.2.0, a *Klebsiella*-dedicated species assignment and genotyping pipeline that uses genome assemblies as input and compares them to a taxonomically curated genome data set (37). To confirm that our genomes belong to the same species, we calculated the genome-wide average nucleotide identity (ANI) for all possible pairs of genomes using fastANI v.1.32 (38). ANI refers to the mean nucleotide identity of all orthologous pairs of genes that are shared between a pair of genomes. We used the recommended ≥95% ANI threshold to delineate species boundary (38). Of the 49 *Klebsiella* isolates identified *in vitro*, only 20 were confirmed as *K. oxytoca sensu stricto* (henceforth referred to as *K. oxytoca*) based on genome sequences. Of the 20 isolates, isolate KPB112 was initially identified as *K. pneumoniae* using the BCID assay. Majority of the non-*K*. *oxytoca sensu stricto* genomes belonged to other species within the *K. oxytoca* species complex: *Klebsiella grimontii* (*n* = 6), *Klebsiella michiganensis* (*n* = 18), *Klebsiella pasteurii* (*n* = 5), whereas another genome initially classified as *K. oxytoca* was later identified as *Klebsiella aerogenes* based on analysis of its genome sequence. Only the 20 confirmed *K. oxytoca* isolates were used for all downstream analysis.

### *In silico* sequence typing and detection of antimicrobial resistance and virulence determinants and plasmid replicons

Sequence types (STs) were identified using mlst v.2.19.0 (https://github.com/tseemann/mlst), which uses previously deposited allele combinations in the *K. oxytoca* PubMLST database (https://pubmlst.org/organisms/klebsiella-oxytoca) (39). The *K. oxytoca* STs are based on sequence variation of seven single copy housekeeping genes (*gapA, infB, mdh, pgi, phoE, rpoB, tonB*) (7). Genome assemblies were screened for the presence of antimicrobial resistance determinants using the AMRFinderPlus v.3.10.23 and its accompanying database of AMR determinants developed by the National Center for Biotechnology Information (NCBI) (40).

We used Kleborate (37) to identify the presence of *Klebsiella pneumoniae*-specific pathogenicity and virulence factors. We also sought to identify the presence of the kleboxymycin biosynthetic gene cluster which encodes the *K. oxytoca* cytotoxins (tilimycin and tilivalline), both of which lead to pathological modifications associated with antibiotic-associated hemorrhagic colitis (5). As previously described (41), the complete kleboxymycin locus from the reference genome (GenBank accession number MF401554.1) was used to query for BLASTn (42) against *K. oxytoca* genomes in this study. Thresholds of >90% nucleotide identity and >90% coverage were used to determine the presence of genes. Genomes having all 12 genes in the biosynthetic gene cluster were recorded as possessing the complete kleboxymycin locus. Visualization of the BLASTn results was done using the BRIG software (43).

We retrieved all the *bla*_OXY-2_ alleles from the BIGSdb *Klebsiella* allele database (https://bigsdb.pasteur.fr/cgi-bin/bigsdb/bigsdb.pl?db=pubmlst_klebsiella_seqdef). A BLASTx search was used to query the database sequences against the *K. oxytoca* genomes. Thresholds of 100% identity and coverage values were used to match an allele, whereas genes with less than 100% identity values were submitted to BIGSdb for curation. We used clinker (https://github.com/gamcil/clinker) to determine the sequence similarity of *bla*_OXY-2_ alleles and generate gene clusters of regions surrounding the *bla*_OXY_ genes. Although *bla*_OXY_ is known to be located in the chromosome, plasmid-encoded *bla*_OXY_ has also been reported in *K. oxytoca* (22). We therefore used the Geneious Prime software v.2022.2.1 to manually inspect the genetic environment of the annotated contigs containing the *bla*_OXY-2_ variants detected to determine if they were associated with mobile genetic elements. Because these were short read data, we used the presence of the gene that encodes the plasmid-encoded Rep initiator protein as an indication of the presence of a plasmid. The presence of plasmids in the genomes was determined by detecting their replicon genes using the PlasmidFinder Database (accessed on November 22, 2022) (44) on ABRicate v.1.0.1 (https://github.com/tseemann/abricate).

### Phylogeny and population structure analysis

Sequence alignments of the 4,535 core genes (i.e., gene families present in 99% of genomes) from the 20 New Hampshire *K. oxytoca* genomes were concatenated to generate the core genome alignment. Single nucleotide polymorphisms (SNPs) were extracted from the core genome alignment using SNP-sites v.2.5.1 (45). The core SNP alignment was used as input for building a maximum likelihood phylogenetic tree using RAxML v.8.2.12 (46) with a generalized time reversible (GTR) (47) model of nucleotide substitution and gamma distribution of rate heterogeneity.

To place our 20 New Hampshire genomes in the global context, we retrieved 1,380 publicly available genomes classified as *K. oxytoca* from the NCBI Isolates Browser (as of August 2022). From these, we narrowed down the data set to include only those sequences from human clinical samples. This yielded a total of 751 genomes. We filtered this data set using the same methods we used for the New Hampshire genomes (i.e., QUAST, CheckM, >200 contigs, N50 <40,000 bp, and ≥95% ANI). To ensure that we included only *K. oxytoca* and not other species of the *K. oxytoca* species complex, we ran Kleborate v2.2.0 and fastANI v.1.32 on these publicly available genomes. Our final non-New Hampshire global data set of *K. oxytoca* consisted of 304 genomes representing 18 countries (including 79 from other parts of the USA) in five continents sampled between 2008 to 2022 (Table S2; Fig. S2). Altogether, this second larger data set consisted of 20 genomes from New Hampshire (sequenced in this study) and 304 publicly available genomes, for a total of 324 genomes representing the global clinical population of *K. oxytoca*. Using SNP-sites v.2.5.1 (45), a total of 167,417 core SNPs were identified from the larger data set. The core SNPs were extracted and used to build a phylogenetic tree using RAxML with the GTR + Gamma model of nucleotide substitution (47). Next, to better understand the evolutionary relationships of *K. oxytoca* lineages from clinical and non-clinical settings, we built another phylogenetic tree consisting of global *K oxytoca* genomes (*n* = 893) from all sources (clinical, environmental, and unknown sources; Table S3). The genomes were filtered the same way as previously described. To build this second global tree, we used the split k-mer distances calculated from assemblies using SKA (Split-Kmer Analysis) (https://github.com/simonrharris/SKA), which benefits from speed and less computational requirement. Split k-mers pertain to any two k-mers in a sequence separated by one or more bases. Phylogenetic trees were visualized and annotated using figtree v.1.4.4 (http://tree.bio.ed.ac.uk/software/figtree/) and Interactive Tree of Life IToL (48).

We built minimum spanning trees using GrapeTree (49) to further examine the genetic relationships of isolates of each of the three most frequently occurring STs in the global data set (ST2, ST176, ST199). These three STs were also detected in the New Hampshire population. A minimum spanning tree represents a set of edges that link nodes by the shortest possible genetic distance and is therefore useful when examining relationships of closely related strains that have undergone short-term divergence (49). We first mapped our genomes to a reference genome (Accession number NZ_OW967518.1 for ST2 genomes, NZ_CP070144.1 for ST176 genomes, and NZ_CP011618.1 for ST199 genomes). We used CSIPhylogeny v.1.4 with default parameters (50) to generate a whole genome SNP alignment. We used Gubbins (v 3.2.1) to remove recombination tracts in the alignments (51). The resulting alignment was used as input in GrapeTree (49).

### Statistical analysis

We carried out all statistical analysis using the stat_compare_means on ggpubr v.0.4.0 package in RStudio v.2022.02.1 + 461. We used Wilcoxon signed rank test to compare the following features between paired groups of ST2, ST176, and ST199: number of accessory genes per genome, number of acquired AMR genes per genome, and number of plasmids per genome. Results were considered significant when *P* < 0.05.

## RESULTS

### Antimicrobial susceptibility and genomic features of bloodstream *K. oxytoca* in New Hampshire

**Fig 1:**
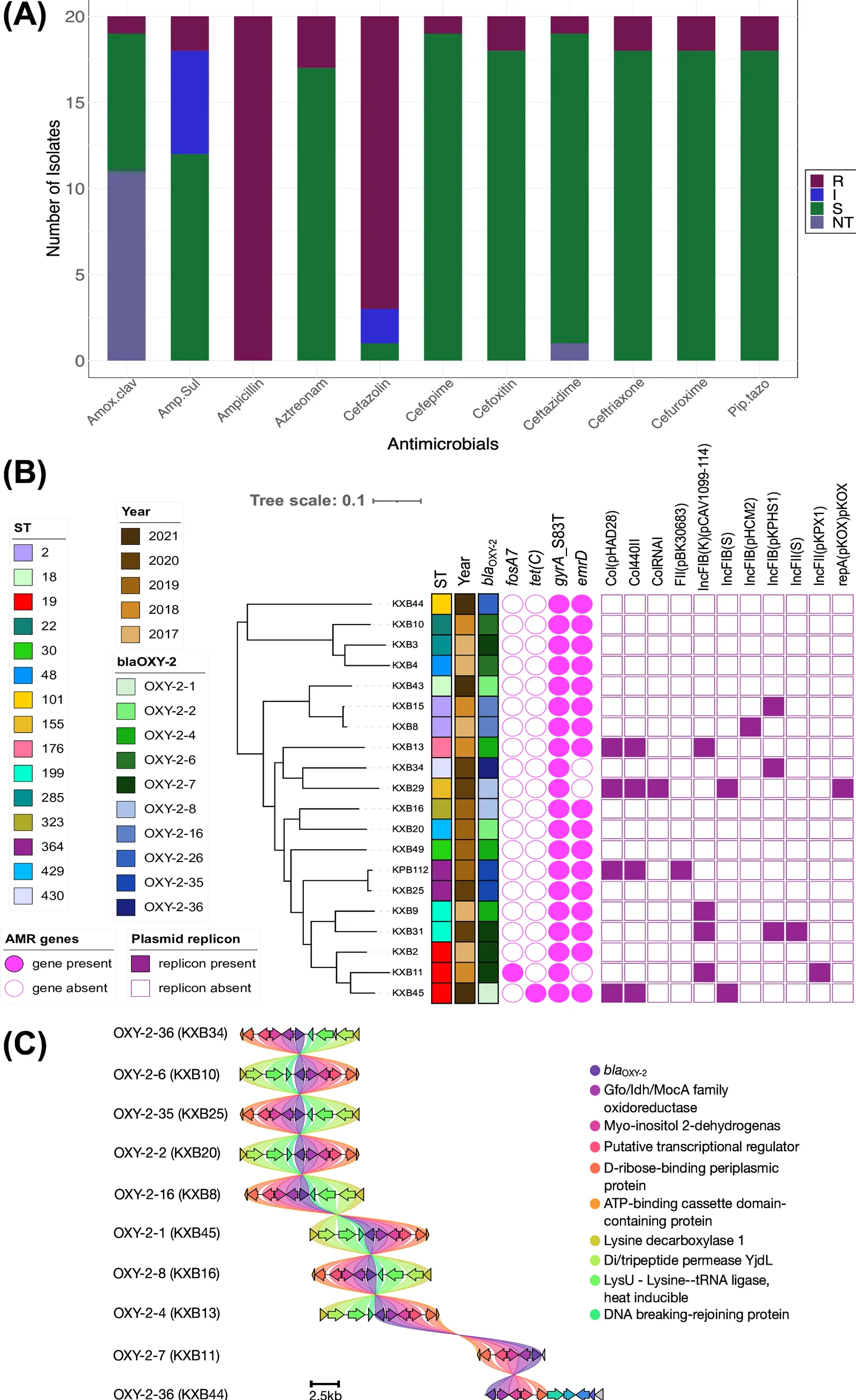
Antimicrobial susceptibility and genomic features of 20 bloodstream isolates of *K. oxytoca* from New Hampshire. (**A**) Results of the *in vitro* antimicrobial susceptibility assay. All isolates were susceptible to ciprofloxacin, ertapenem, gentamicin, levofloxacin, meropenem, sulfamethoxazole/trimethoprim (SXT), amikacin. Color legend: R = resistant, I = intermediate resistance, S = susceptible, NT = not tested. Amox.clav = amoxicillin and clavulanic acid. Amp.Sul = ampicillin and sulbactam. Pip.tazo = piperacillin and tazobactam. Details are shown in Table S1. (**B**) Phylogenetic relationship of the 20 bloodstream *K. oxytoca* isolates. The midpoint-rooted maximum likelihood phylogenetic tree is based on sequence alignment of 4,535 concatenated core genes. Tree scale represents the number of substitutions per site. The phylogeny is annotated to show sequence type (ST), year of isolation, *bla*_OXY-2_ variants, AMR determinants, and plasmid replicon types. (**C**) Genetic neighborhood of the *bla*_OXY-2_ variants. Gene families are represented by the same-colored arrows and the encoded proteins are shown in the color legend. Black lines connecting colored arrows between the *bla*_OXY-2_ variants indicate the sequence similarity. The representative genomes used for comparison are shown in parenthesis.

We obtained 20 confirmed *K. oxytoca* isolates from clinical blood culture specimens submitted to DHMC, New Hampshire, USA from February 2017 to November 2021. Using 20 antimicrobial compounds tested against *K. oxytoca in vitro*, all 20 isolates exhibited resistance to at least one antimicrobial agent (Fig. 1A; Table S1). As expected, all isolates were resistant to ampicillin, whereas 17 isolates were resistant to cefazolin. The median number of antimicrobials (excluding ampicillin) to which the genomes were resistant was one, whereas isolates KXB49 and KXB44 exhibited resistance to nine and eight antimicrobial agents (excluding ampicillin), respectively. We observed intermediate resistance to ampicillin-sulbactam in six isolates. No demonstrable resistance to aminoglycosides, antifolate, carbapenems, and fluoroquinolones was observed in any isolate.

*De novo* genome assembly produced sequences ranging from 5.7 to 6.1 Mbp in length (Table S2). Annotation of assembled genomes revealed a total of 10,236 families of orthologous genes, which can be classified into 4,535 core genes (genes present in ≥99% of genomes), 230 soft core genes (present in 95 to <99% of genomes), 1,106 shell genes (present in 15 to <95% of genomes), and 4,365 cloud genes (present in <15% of genomes) (Table S4). The number of genes per genome ranged from 5,245 (isolate KXB2) to 5,740 (isolate KXB45) (Table S4).

Multi-locus sequence typing (MLST) analysis revealed the presence of 15 STs, including two novel STs (ST429 in isolate KXB20 and ST430 in isolate KXB34) (Fig. 1B; Table S5). We did not observe a single ST that dominated the New Hampshire population. STs represented by more than one isolate included ST19 (three genomes), ST2 (two genomes), ST199 (two genomes), and ST364 (two genomes). All other STs were represented by a single isolate. Five STs in New Hampshire (ST2, ST19, ST199, ST155, ST176) are members of the clonal complex 2, which has been associated with carbapenem resistance and outbreaks worldwide (5, 41). We did not detect phylogenetic structuring of STs based on the year of isolation.

We sought to identify the presence of AMR determinants in the New Hampshire genomes (Fig. 1B; Table S5). All 20 genomes harbored the gene *bla*_OXY-2_ encoding the extended-spectrum class A beta-lactamase. Its presence is consistent with the known intrinsic carriage of *bla*_OXY_ in the *K. oxytoca* complex (21). At constitutive low levels, OXY beta-lactamases confer resistance to aminopenicillins and carboxypenicillins (52). The presence of *bla*_OXY-2_ is consistent with the *in vitro* ampicillin susceptibility test results of the 20 isolates. At highly induced levels, OXY beta-lactamases confer resistance to penicillins, cephalosporins, and monobactams (52). We observed this to be true in isolates KXB44 and KXB49, which both exhibited high *in vitro* resistance against ampicillin, ampicillin-sulbactam, cefazolin, cefoxitin, ceftriaxone, cefuroxime, and aztreonam (Table S1), suggesting the overproduction of the OXY beta-lactamase (21, 53) in these isolates. However, we did not detect the known mutations in the *bla*_OXY_ promoter −10 and −35 motifs, as previously described (5, 41, 54).

We also detected the presence of genes mediating resistance to fosfomycin (*fosA*; present in one genome), and tetracycline (*tetC*; present in one genome). The multidrug efflux pump encoded by *emrD,* which exports a wide range of hydrophobic compounds out of the cells (55, 56), was also detected in 17 genomes. In addition to intrinsic (*oqxA/B*) and acquired AMR genes, we also detected the non-synonymous point mutation *gyrA*_S83T in all 20 genomes. This mutation is associated with reduced susceptibility to quinolone as a result of the diminished binding of quinolones to the topoisomerase-DNA complex (26).

We identified ten variants of *bla*_OXY-2_ (Fig. 1B). Different *bla*_OXY-2_ variants were interspersed across the core genome phylogeny and the same *bla*_OXY-2_ variant was represented by different STs. The most common variant was *bla*_OXY-2-7_, which was detected in four genomes across ST19, ST199, and ST22. We detected two novel *bla*_OXY-2_ variants, which were assigned *bla*_OXY-2-35_ (KXB25 and KPB112) and *bla*_OXY-2-36_ (KXB34) in the BIGSdb *Klebsiella* allele database. Analysis of the genetic neighborhood of the *bla*_OXY-2_ variants revealed structural heterogeneity characterized by gene rearrangements and the presence or absence of certain flanking genes (Fig. 1C). The *bla*_OXY-2_ gene is frequently flanked by genes encoding the Gfo/Idh/MocA family oxidoreductase, myo-inositol 2-dehydrogenase, putative transcriptional regulator, and D-ribose-binding periplasmic protein. None of the *bla*_OXY-2_ variants in the New Hampshire genomes was located on a plasmid.

We also sought to examine the presence of plasmid replicons in the genomes. We detected a total of 11 different types of plasmid replicons in the New Hampshire *K. oxytoca* genomes (Fig. 1B; Table S5). Plasmid replicons Col(pHAD28), Col440II, and IncFIB(K)(pCAV1099-114) were most frequently detected (four genomes for each type). Half of the isolates (*n* = 10) harbored at least one plasmid replicon in their genomes. Strain KXB29 had the highest number of plasmid replicons (*n* = 5) detected in its genome, whereas three plasmid replicons each were detected in KXB3, KXB13, KXB45, and KPB112.

**Fig 2:**
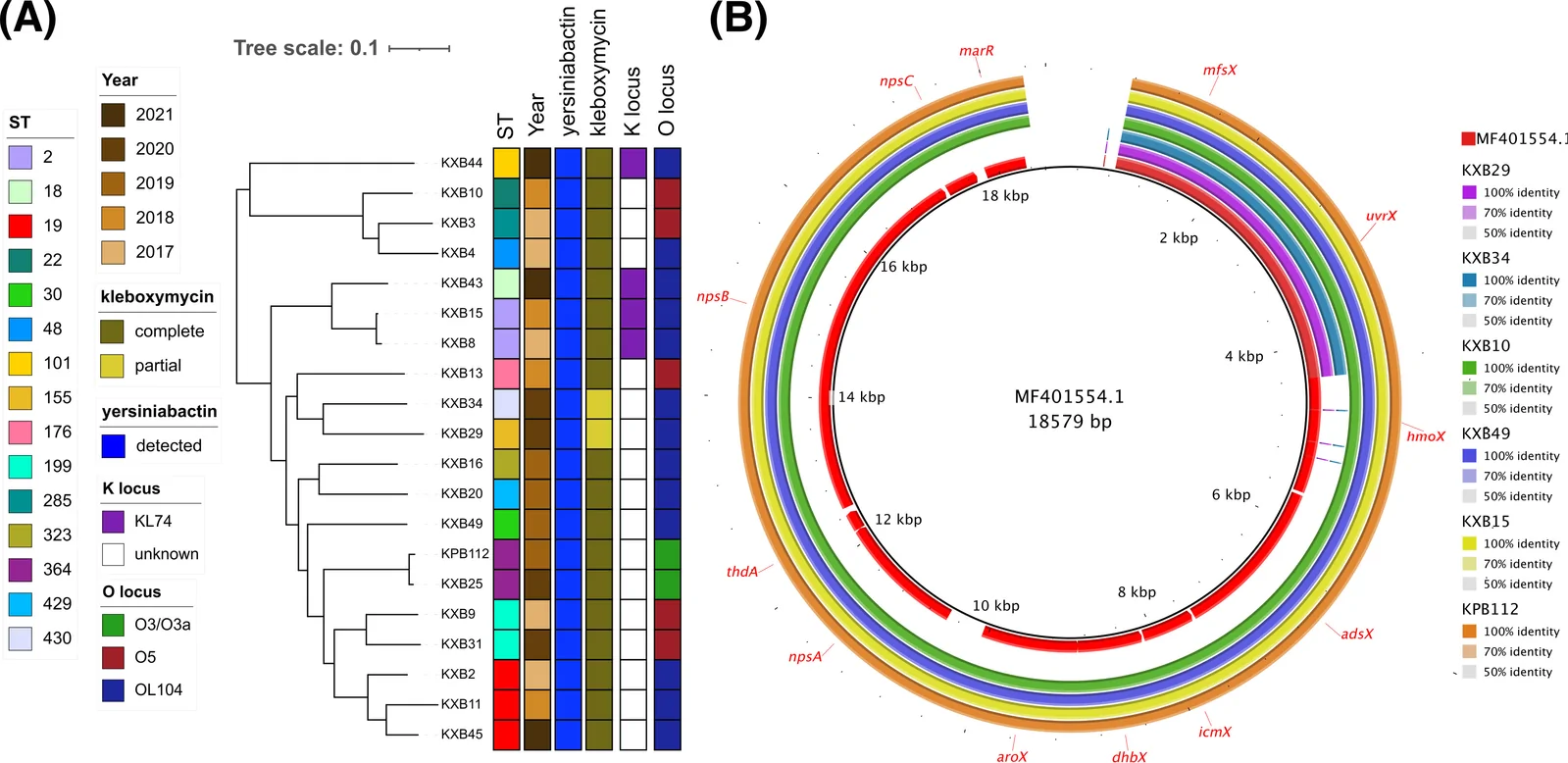
Virulence determinants detected in 20 bloodstream *K. oxytoca* isolates from New Hampshire. (**A**) A midpoint-rooted phylogenetic tree showing the sequence types (STs), year of isolation, K antigen, O antigen, and yersiniabactin and kleboxymycin loci. The tree is identical to that in Fig. 1B. (**B**) Genomic map showing sequence similarities of the kleboxymycin gene cluster from the reference genome (GenBank accession number: MF401554.1) when compared to representative genomes from this study harboring the complete (KXB15, KXB49, and KPB112) and partial (KXB29 and KXB34) gene cluster.

Among the *Klebsiella pneumoniae*-specific virulence determinants screened for by the Kleborate tool, only the yersiniabactin *ybt* locus was detected. This locus was detected in all the New Hampshire genomes (Fig. 2A). Yersiniabactin plays an important role in bacterial survival in the blood (57). We also detected the presence of the complete kleboxymycin locus in 18 genomes, whereas two genomes KXB29 and KXB34 only possessed two complete genes *mfsX* (which encodes a multidrug efflux major facilitator superfamily transporter) and *uvrX* (which encodes excinuclease ABC subunit UvrA) (5), from the kleboxymycin gene cluster (Fig. 2B). We also detected the presence of the capsular polysaccharide (K antigen) and outer lipopolysaccharide (containing O antigen), which are major surface antigens of *K. pneumoniae* (58) but are poorly studied in *K. oxytoca* species complex. Within the New Hampshire *K. oxytoca*, four genomes carry the type KL74, whereas the rest are of unknown K types. We also identified three O antigen types: O3/O3a (*n* = 2 genomes), O5 (*n* = 5 genomes), and OL104 (*n* = 13 genomes) (Fig. 2A).

### The global population of clinical *K. oxytoca* is phylogenetically diverse

**Fig 3:**
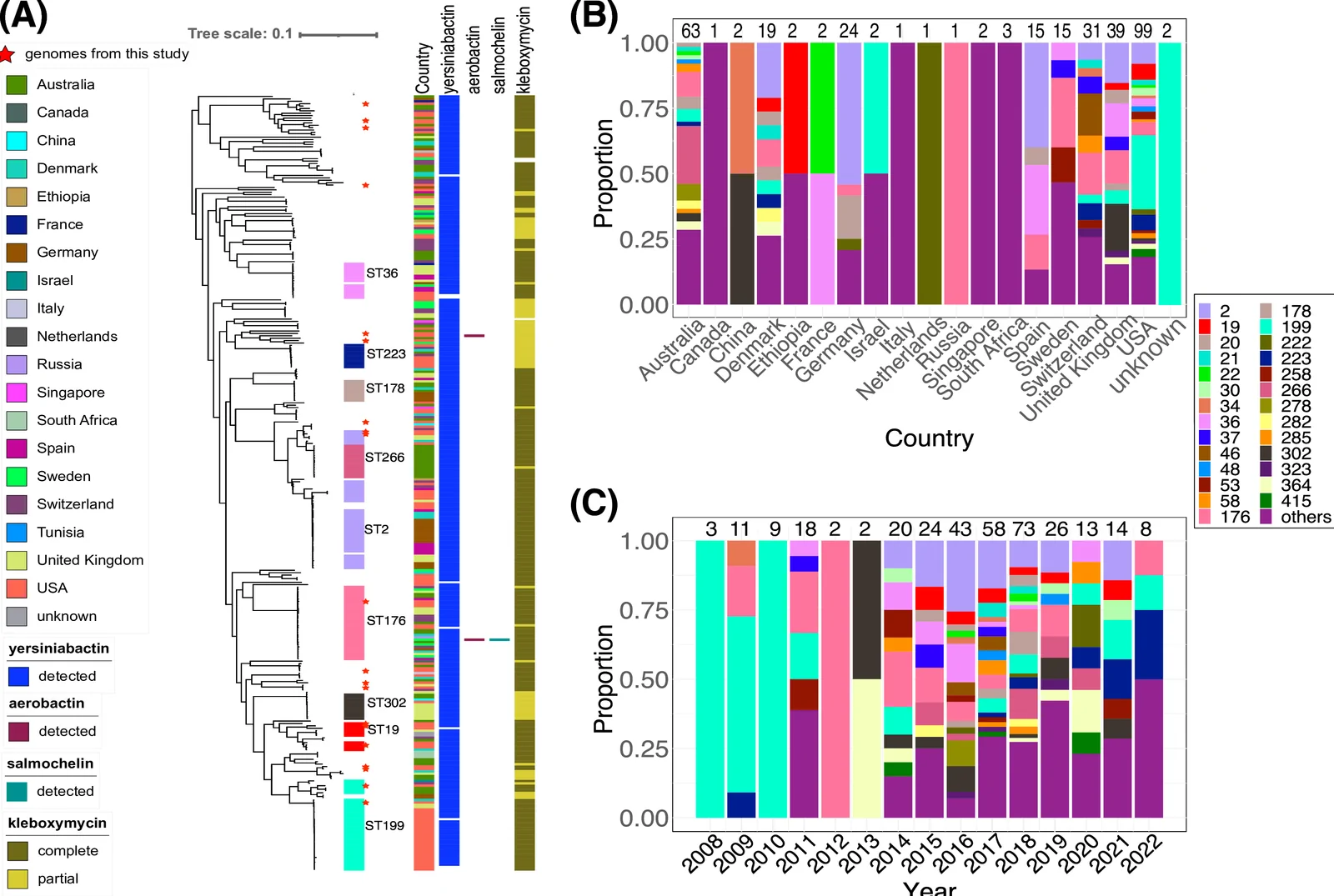
Phylogenetic relationship and global population structure of clinical *K. oxytoca* (*n* = 324 genomes). (**A**) A midpoint-rooted phylogenetic tree showing the country of origin and presence of virulence determinants. The tree was built on the sequence alignment of 4,062 concatenated core genes. Tree scale represents the number of substitutions per site. Branches with red stars are used to indicate the 20 New Hampshire genomes sequenced in this study. For visual clarity, only the most common sequence types (STs) are labeled in color on the phylogeny. (**B**) Distribution and proportion of *K. oxytoca* genomes belonging to different STs by year of collection. (**C**) Distribution and proportion of *K. oxytoca* belonging to different STs by country of isolation. For panels B and C, the total number of genomes per year and per country is shown at the top of the bars. STs with less than three genomes are included in the category “Others.”

To place the New Hampshire *K. oxytoca* onto a broader context, we compared them to 304 publicly available genomes of clinical isolates from 18 countries (including 79 from other parts of the USA) across five continents sampled between 2008 and 2022 (Fig. 3A). In total, this larger data set included 324 genomes, consisting of the 20 New Hampshire genomes and 304 global non-New Hampshire genomes. Calculation of the genome-wide ANI among all genome pairs in this larger data set showed ANI values ranging from 98.51% to 100% (Fig. S3; Table S6). Pan-genome analysis of this larger clinical data set identified 35,625 genes categorized into 4,062 core genes, 399 soft core genes, 1,413 shell genes, and 29,751 cloud genes (Table S7). Phylogenetic analysis of this larger data set based on the sequence variation of 4,062 core genes showed that the New Hampshire genomes are widely distributed within the global population of clinical *K. oxytoca* (Fig. 3A).

Based on the 7-gene MLST assignment (7), we identified 65 known STs and 33 genomes with novel MLST allele configuration (representing 10.18% of the data set) (Table S8). Three STs dominated the global clinical *K. oxytoca* population: ST2 (*n* = 39 genomes representing 12.03% of the data set), ST176 (*n* = 31 genomes, 9.56%), and ST199 (*n* = 36 genomes, 11.11%), which all together represented 32.71% of the entire data set. ST2 is found in six countries (Denmark = 4, Germany = 13, Spain = 6, Switzerland = 2, United Kingdom = 6, USA = 8). ST176 is found in nine countries (Australia = 6, Denmark = 2, Germany = 1, Russia = 1, Spain = 2, Sweden = 4, Switzerland = 5, United Kingdom = 5, USA = 5). ST199 is found in six countries (Australia = 3, Denmark = 1, Israel = 1, Switzerland = 1, United Kingdom, USA = 28) (Fig. 3B). These three STs were frequently but intermittently detected throughout the sampling period of the global clinical data set: ST2 from 2014 to 2019 and 2021; ST176 in 2009, 2011, 2014–2019, 2022; and ST199 in 2008–2011, 2014, 2017, 2018, 2020–2022 (Fig. 3C). Note that we also observed ST variants that differ in one to two loci from the major STs. For instance, ST2, ST266, and ST415 (unlabeled) differ by a single locus (*phoE*), whereas ST199 and the unlabeled ST differed in two loci (*mdh* and *phoE*). A total of 38 STs were represented by three or fewer genomes. The low-frequency STs were also widely distributed across 10 of the 15 years of sampling and across 14 of the 18 countries included in this data set. We also detected phylogenetic structuring of a few STs based on the geographical source of the isolates. For example, all ST266 were from Australia, whereas ST2 is dominant in Germany (*n* = 13/39 genomes), ST199 in the USA (*n* = 25/36 genomes), and ST302 in the United Kingdom (*n* = 7/11 genomes).

We further sought to elucidate the relationship of the New Hampshire *K. oxytoca* within the global population from clinical and non-clinical sources. To accomplish this, we built a phylogenetic tree based on split k-mer distances calculated from the core genome alignment. This second global data set included genomes of *K. oxytoca* isolates from clinical, environmental, and unknown sources (*n* = 893) genomes (Table S3). To validate the utility of the split k-mer approach, we first used it to reconstruct a phylogeny of the first global data set (i.e*.*, global clinical genomes only; *n* = 324 genomes). We observed topological concordance (Fig. S4A) and a strong correlation (*R* = 1, *P* < 2.2e-16; Fig. S4B) between the trees built from the split k-mers and the core genome SNPs. Next, we used the split k-mers to build the second global phylogeny consisting of *K. oxytoca* from clinical and non-clinical sources. Results from this second global tree recapitulated what we observed in the clinical population in terms of the diversity of the STs and the intermingling of the New Hampshire genomes within the global population (Fig. S5). However, it is important to note that this second global data set heavily favors clinical isolates and that numerous isolates do not have information about their sampling source.

### The global population of clinical *K. oxytoca* carries a diverse suite of AMR determinants

**Fig 4:**
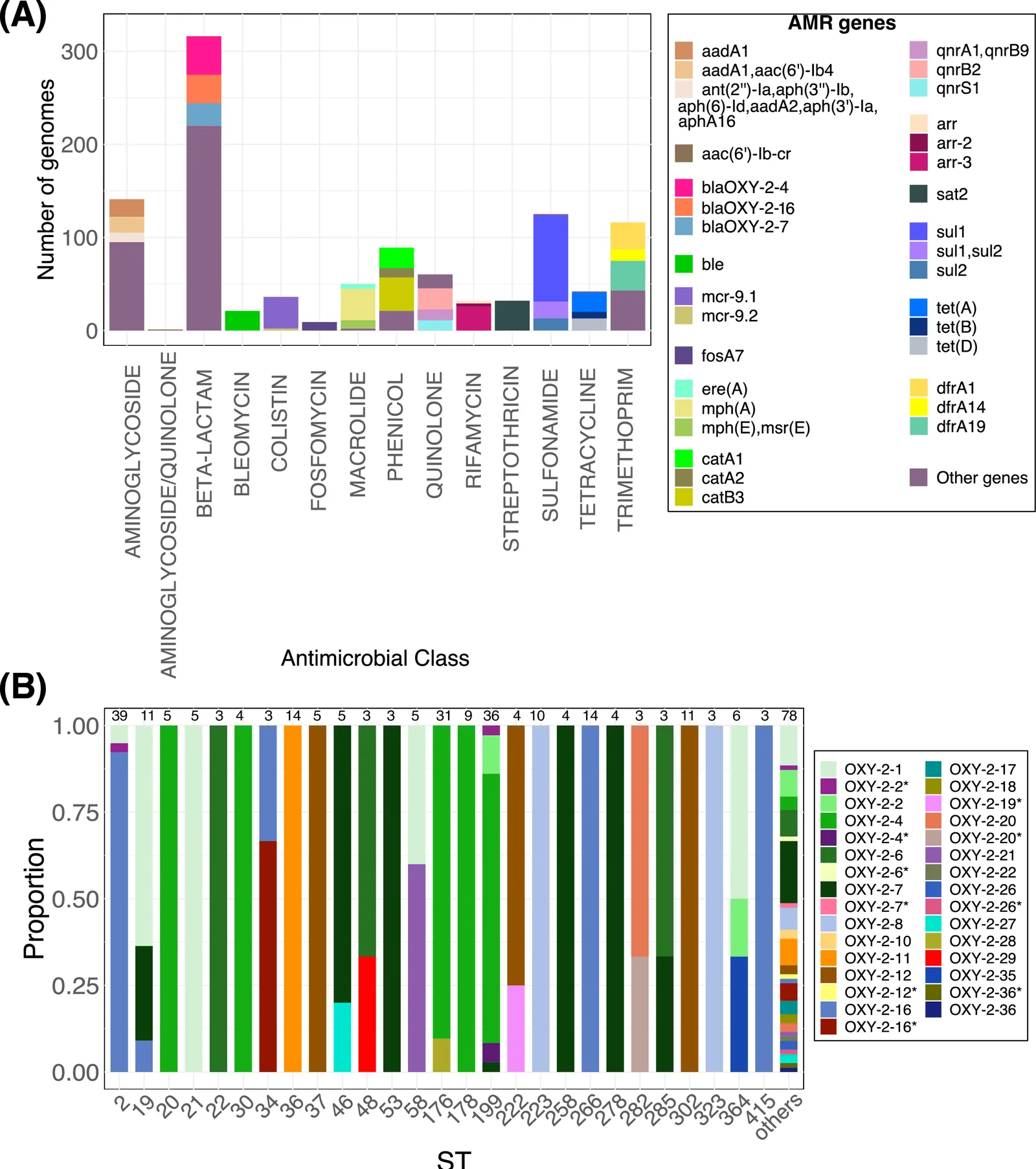
Distribution of antimicrobial resistance (AMR) determinants in the global clinical *K. oxytoca* population (*n* = 324 genomes). (**A**) Number of genomes harboring genetic determinants to resistance against 15 antimicrobial drug classes. Colors of bars represent the three most frequently occurring genes per antimicrobial class, whereas other less commonly occurring genes are denoted as “Other genes.” Multiple genes grouped together suggest that they co-occur at the same frequency in the data set. (**B**) Distribution and proportion of *bla*_OXY-2_ variants per sequence type (ST). Only STs that are represented by at least three genomes are shown. Genes not having 100% sequence similarity with *bla*_OXY-2_ genes in the databases are indicated by an asterisk.

The global population of clinical *K. oxytoca* (*n* = 324 genomes) harbors a remarkably diverse assortment of AMR determinants (Fig. 4A; Table S7). *In silico* detection of acquired AMR genes in individual genomes revealed the presence of 151 AMR genes conferring resistance to 14 antimicrobial classes, with the novel *bla*_OXY-2_ alleles from the global clinical data set being counted as one gene. On average, an individual genome possessed six AMR genes. The genome with the highest number of AMR genes (*n* = 25) came from Denmark sampled in 2015. We also found high number of genomes carrying at least one gene conferring resistance to aminoglycosides (141 genomes), sulfonamides (125 genomes), and trimethoprim (116 genomes). We also detected the presence of AMR determinants to last resort antimicrobials such as carbapenems and colistin in the global population of clinical *K. oxytoca* genomes. Carbapenemases detected in this data set included *bla*_KPC_ (*n* = six genomes representing 1.86% of the data set), *bla*_NDM_ (*n* = 21 genomes, 6.48%), *bla*_IMP_ (*n* = 13 genomes, 4.01%), and *bla*_VIM_ (*n* = 19, 5.86%). A total of 36 genomes (representing 11.11% of the data set) harbored either one of the colistin resistance genes *mcr9.1* (*n* = 34) and *mcr9.2* (*n* = 2). Furthermore, over half of the genomes (61.76%, *n* = 21) harboring the colistin resistance gene *mcr*9.1 belonged to ST199 clone and were from the United States.

We detected a total of 21 *bla*_OXY-2_ variants in the global clinical data set (Fig. 4B), of which 10 did not exhibit 100% sequence similarity to known *bla*_OXY-2_ genes (indicated by asterisks in Fig. 4B; Table S8). Except the *bla*_OXY-2-35_ and *bla*_OXY-2-36_, the *bla*_OXY-2_ variants detected in our local New Hampshire genomes were also present in several genomes in the global clinical data set. The most frequently occurring *bla*_OXY_ alleles were *bla*_OXY-2-4_ (*n* = 77 genomes, representing 23.7% of the data set), *bla*_OXY-2-16_ (*n* = 56 genomes, 17.3%) and *bla*_OXY-2-7_ (*n* = 34 genomes, 10.5%). Next, we aimed to determine the distribution of *bla*_OXY_ alleles across different STs. Here, we only limited our analysis to those STs consisting of ≥3 genomes. We observed that some alleles were more commonly detected in certain STs. For instance, *bla*_OXY-2-16_ is dominant in ST2 (*n* = 36/39 genomes) and present in all ST266 (*n* = 14) genomes. The allele *bla*_OXY-2-16_ was observed to be common in ST176 (*n* = 28/31 genomes) and ST199 (*n* = 28/36 genomes) genomes. We did not observe any structure in the distribution of *bla*_OXY-2_ across the various countries of isolation; however, *bla*_OXY-2-4_ appears to be prevalent between 2009 and 2011 (Fig. S7). Beginning 2014, a more diverse *bla*_OXY-2_ allele can be detected, including the presence of *bla*_OXY-2-16_ and *bla*_OXY-2-21_. However, caution must be considered when expounding on these results as sampling biases across the wide temporal-spatial planes may play an important role in the interpretations.

We also examined the diversity of plasmids and virulence determinants in the global clinical *K. oxytoca* population. A total of 54 plasmid replicon types were detected. The most frequent plasmid replicons were Col(pHAD28) and Col440II detected in 110 (representing 33.9%) and 109 genomes (33.6%), respectively (Fig. S7; Table S8). The yersiniabactin locus was detected in 315 genomes, representing 97.2% (Fig. 3; Table S8). Genomes lacking yersiniabactin locus belonged to ST19 (one genome), ST36 (two genomes), ST199 (three genomes), ST176 (one genome), and ST375 (one genome) from the United States and ST 96 from Denmark (one genome). The aerobactin and salmochelin virulence loci were detected in two and one genome(s), respectively. Similar to yersiniabactin, aerobactin and sideophores are known to enhance bacterial survival in the blood by improving the sequestration of iron (57). The complete kleboxymycin biosynthetic gene cluster was detected in 251 genomes (representing 77.5%), whereas it was absent in 0.9% (*n* = 3) of the genomes (Fig. 3; Table S8).

### Genomic features of the three dominant STs in the global clinical population

**Fig 5:**
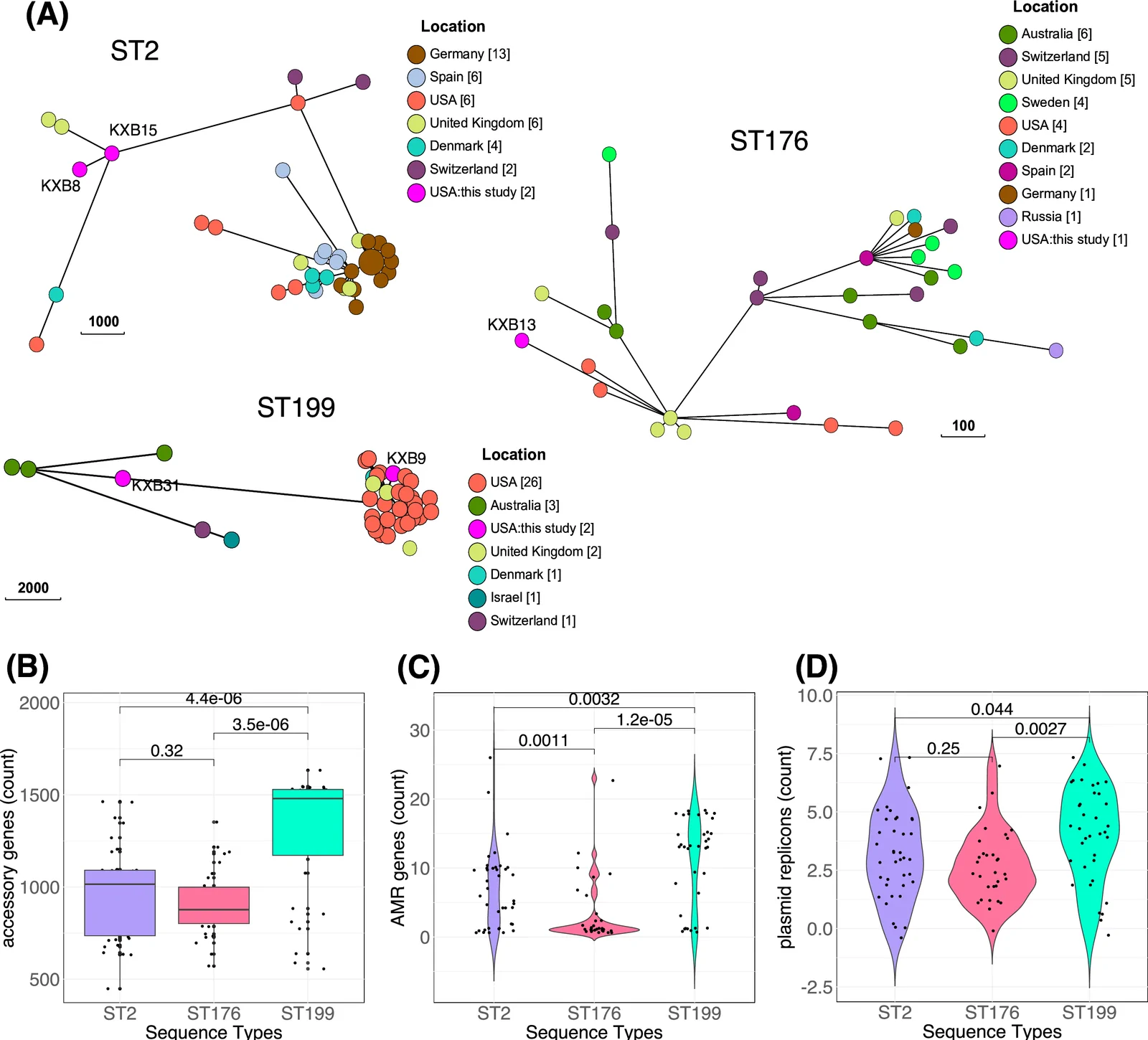
Phylogenetic relationship and genomic comparison of ST2, ST176, and ST199 in the global clinical *K. oxytoca* population. (**A**) Minimal spanning trees of *K. oxytoca* ST2, ST176, and ST199 based on allelic variation in the whole genome SNP alignments. Nodes represent isolates with distinct allelic profiles. The nodes are colored by their country of origin. The number of isolates from each sequence type from each country is indicated in brackets. The scale represents number of SNPs and the length of the scale is proportional to the number of SNP differences. (**B**) Boxplots showing the total number of accessory genes per genome at 25th, 50th, and 75th percentiles. Black dots outside of the boxplot show the number of accessory genes outside of the interquartile range. Black horizontal line inside the box represents the median. Violin jitter plots showing the number of (**C**) AMR genes per genome and (**D**) plasmid replicons per genome. For all panels, Wilcoxon test was used to compare paired groups of ST2, ST176, and ST199.

The three most common STs in the global clinical population were ST2, ST176, and ST199, which were also present in the New Hampshire population. We therefore sought to compare the genomic features and inter-strain relationships within each ST to understand how certain genetic features shape members of this population. First, we built recombination-free minimum spanning trees using the whole genome SNP alignment of each ST as input (Fig. 5A). In ST2, we observed a cluster of genomes originating from Germany, United Kingdom, Spain, Denmark, and other parts of the United States (Cincinnati—two genomes, Pittsburgh—three genomes, unknown state—one genome) that vary in ≤316 SNPs. However, the two ST2 genomes from New Hampshire (KXB8 and KXB15) were distantly related to the members of this cluster, with SNPs ranging from 6,305 to 6,386, and were closely related to two other genomes from the United Kingdom. In ST199, we observed a cluster with ≤443 SNPs between the genomes from the United States (from New Hampshire [KXB9] and other states in the USA: Pittsburgh—one genome, Virginia—22 genomes, and unknown state—three genomes), United Kingdom, and Denmark. However, the other New Hampshire genome (KXB31) was distantly related from this cluster. The large genetic distances among ST2 genomes and among ST199 genomes from geographically disparate locations may indicate non-recent cross-country transmission events. In contrast, there was no observable clustering of any subset of the ST176 genomes regardless of geographic origin, with strains separated by distances of up to 434 SNPs.

There were significant differences in terms of accessory genes among the three STs. Significant differences were observed in the total number of accessory genes between ST2 and ST199 as well as between ST176 and ST199 (both *P* < 0.001, Wilcoxon signed rank test; Fig. 5B). The three STs significantly differed in the number of acquired AMR determinants carried per genome (*P* < 0.05, Wilcoxon signed rank test; Fig. 5C). We also did not detect any instance of AMR genes unique to each ST. However, some AMR genes were more frequently detected in certain STs (Table S8). For instance, the most common aminoglycoside resistance gene in ST2 genomes was *aadA1* (*n* = 26/39 genomes), whereas ST199 genomes often carried *aph(3')-Ia* (*n* = 25/36 genomes), *aadA2* (*n* = 21/36 genomes), and *ant(2'')-Ia* (*n* = 20/36 genomes). Genes associated with resistance to aminoglycosides were sparsely present in ST176 genomes. ST199 genomes most frequently harbored beta-lactamases (*bla*_KPC_ = 22 genomes, *bla*_OXA-2_ = 20 genomes, *bla*_SHV-30_ = 16 genomes) and colistin resistance gene (*mcr*-9.1 =21 genomes). The number of plasmid replicons per genome differed significantly between ST2 and ST199 as well as between ST176 and ST199 (both *P* < 0.05, Wilcoxon signed rank test; Fig. 5D). Plasmid replicons belonging to incompatibility group IncFII(S) (*n* = 23 genomes), IncM1 (*n* = 23 genomes), and IncFII(SARC14) (*n* = 22 genomes) were most common in ST199 genomes, whereas IncFIB(K) (*n* = 20) was most common in ST2. There were no plasmid replicon types that were unique to ST176 genomes.

Lastly, we also examined the presence of chromosomal mutations associated with reduced susceptibility to antimicrobials (Table S8). The most common mutations were those conferring quinolone resistance. The mutation *gyrA*_S83T was frequently detected in ST176 (*n* = 28/31 genomes) and ST199 (*n* = 36/36 genomes), whereas *gyrA*_S83I was commonly detected in ST2 (*n* = 25/39 genomes). The mutation *ompk*36_D135DGD conferring carbapenem resistance was detected only in members of ST2 (*n* = 9/39 genomes).

## DISCUSSION

Multidrug-resistant *K. oxytoca* poses an emerging threat in many healthcare settings. In this study, we report the phenotypic and genomic features of *K. oxytoca* recovered from bloodstream infections in a single hospital in New Hampshire, USA and placed them in the wider clinical population from 18 countries. At both geographical scales, clinical *K. oxytoca* is phylogenetically diverse, harboring AMR genes including several *bla*_OXY-2_ variants. In the hospital population of *K. oxytoca* in New Hampshire, multiple distinct lineages carry similar AMR determinants, indicating that the distribution of AMR is independent of the genetic background. The global *K. oxytoca* population is genetically diverse, but there is evidence for wide dissemination of a few lineages carrying distinct set of AMR determinants.

As we have observed in our study, identification of *K. oxytoca sensu stricto* using the FilmArray BCID/BCID2 assay was accurate in less than half of the isolates, albeit the majority of species assignments were to members of the *K. oxytoca* species complex. Shortfalls associated with the use of various phenotypic, biochemical, and PCR-based tests for differentiating members of the *K. oxytoca* species complex (5) suggest that any possible differential clinical significance between species within this complex is poorly understood and their prevalence largely unknown. Development of species-specific ribosomal subunit protein markers for MALDI-TOF has shown to improve accuracy and robustness in the identification of *Klebsiella* spp. (59). Precise identification of bacterial species is not only important for improving patient care (as this may impact antimicrobial susceptibility test interpretation, patient treatment, and prognosis prediction), but also improves our understanding of its epidemiology. The use of whole genome sequencing offers a significantly improved resolution and granularity to distinguish members of the *K. oxytoca* species complex. The core genes we identified are an excellent starting point for developing a genome-based taxonomy of *K. oxytoca* lineages, which will be particularly useful for monitoring pathogen evolution and resistance transmission (5, 60). The use of core genome MLST schemes have been demonstrated to provide fine scale resolution for strain discrimination in *K. pneumoniae* (61).

Emerging opportunistic pathogens like *K. oxytoca* harboring genetic determinants that confer resistance to 14 antimicrobial classes and are brought about by different mechanisms (intrinsic, acquired, point mutations) are a cause for concern. The remarkable diversity of the intrinsic beta-lactamases encoded by *bla*_OXY-2_ and the independent acquisition of other AMR determinants across the global population need to be closely monitored. There are currently 35 *bla*_OXY-2_ variants in the Institut Pasteur *Klebsiella* database (as of April 2023). Our analysis of the global *K. oxytoca* ascertains that many more *bla*_OXY-2_ variants remain to be discovered. Although the *bla*_OXY_ gene within *K. oxytoca* species complex has been evolving for the past 100 million years, its evolution has not been associated with phenotypic changes in antimicrobial resistance (21). However, we do stress the need for a more exhaustive investigation as the predominantly chromosomally encoded gene (*bla*_OXY-2_) has also been detected on a plasmid (22). Moreover, that the global population already carries numerous AMR determinants against last resort antimicrobials such as carbapenems and colistin is particularly worrisome. Although we observed no more than 10% of the isolates carrying the genes conferring resistance to carbapenems and colistin, their presence in mobile genetic elements can rapidly and widely disseminate them to other strains and species.

Our study reveals the growing importance of lineages belonging to the international clonal complex 2, which has been responsible for several outbreaks (5, 62). This lineage has been described as a potential high risk with the propensity for dissemination of AMR (5, 63). In our study, clonal complex 2 genomes belonging to ST2, ST176, and ST199 (5, 41, 62) made up a third of the global data set. Although genotyping and diversity studies of *K. oxytoca* are scarce, previous reports reveal numerous STs were already circulating in multiple countries, but these three lineages were not particularly prevalent. In 14 hospitals across Europe and Israel (sampled in 2008–2011), a total of 34 STs were identified in 68 rectal carriage isolates and were to be non-susceptible to extended-spectrum cephalosporins (62). Of these isolates, 82% belonged to STs that make up clonal complex 2 (ST2, ST9, ST36, ST88, ST141) (62). In neonates in Connecticut, USA (sampled in 2013–2018), ST173 and ST246 were identified in six *K. oxytoca* isolates associated with necrotizing enterocolitis (11). In Poland (sampled in 2009–2014), ST145, ST146, and ST166 were identified in 23 clinical isolates producing VIM/IMP-type metallo-beta-lactamases (64). A total of 13 STs, including ST2, were identified in 18 isolates of extended-spectrum-beta-lactamase-producing *K. oxytoca* from 14 Dutch hospitals (sampled in 2011–2014) (65). In 92 clinical isolates from Australia (sampled in 2018–2019), four ST176 and three ST199 isolates were present among 26 STs, but no ST2 was detected (41). The only study that reported evidence of a clonal expansion was that of ST2 in 20 hospitals across the United Kingdom and Ireland (sampled in 2001–2010), where 41 multidrug-resistant *K. oxytoca* isolates from bloodstream infections were recovered (66). In our analyses of the global *K. oxytoca* data set spanning 15 years and 18 countries, we show that ST2, ST176, and ST199 are prevalent and widely disseminated in clinical infections. The success of these lineages may have been influenced by the unique repertoire of accessory genes, AMR determinants, and plasmids that each ST carries. Long-term and geographically broader surveillance of these three lineages is critical to effective disease control, therapeutic options, and international efforts to curb the spread of AMR.

Our study benefits from the analyses of both hospital-level and global populations of clinical *K. oxytoca* that allowed us to gain a comprehensive perspective of the standing diversity. Nonetheless, we recognize the limitations of our study. First, the New Hampshire isolates only included those recovered from bacteremia cases and not from other infections. As *K. oxytoca* is also implicated in a wide range of diseases (3, 5, 9 – 11, 14), other STs and AMR determinants associated with non-bacteremia infections were missed in our analyses. *K. oxytoca* from contaminated hospital equipment, fomites, and environments (e.g*.*, sinks, gloves) are also important sources for *K. oxytoca* sampling (67 – 69). The characterization of the local *K. oxytoca* population is incomplete without the inclusion of other diseases and environments where *K. oxytoca* is known to reside. Second, the global data set is biased toward certain countries where more sampling or reporting has been carried out (e.g*.*, USA, Australia), while certain continents were poorly represented (e.g*.*, one country in Asia, two countries in Africa, no country from South America). A broader surveillance and whole genome sequencing of *K. oxytoca* from geographically and ecologically diverse sources, including carriage, will likely reveal previously unrecognized phylogenetic lineages, *bla*_OXY-2_ variants, AMR determinants, and surface antigen types. We also acknowledge that the frequency of resistance determinants in the global data set may not be overall reflective of the clinical ecosystem, as publicly released genomes may arise from studies favoring detection of multidrug resistance in bacteria. Nonetheless, our work provides a solid foundation to future investigations of *K. oxytoca* diversity, epidemiology, and evolution.

In summary, our study highlights the successful dissemination and prevalence of AMR-harboring lineages of clinical *K. oxytoca* occurring over different geographical scales. We also show that the application of whole genome sequencing in disease surveillance is instrumental in accurate species identification and detection of specific AMR determinants. Our findings have important implications for monitoring the emergence and evolution of antimicrobial resistant clones of clinical *K. oxytoca,* as part of international initiatives and local epidemiological efforts for the tracking and mitigation of AMR.

## Data Availability

Genome sequence data of the 20 New Hampshire *K. oxytoca* isolates sequenced in this study have been deposited in the NCBI Sequence Read Archive under BioProject accession number PRJNA908117. MLST sequences of isolates KXB20 and KXB34 with novel ST configurations were submitted to the pubMLST database and were given new ST designations (ST429 and ST430, respectively). Sequence data of the 304 globally distributed non-New Hampshire *K. oxytoca* are publicly available in NCBI. Accession numbers of the 20 New Hampshire genomes sequenced in this study and the genomes retrieved from NCBI are listed in Tables S2 and S3. The 20 New Hampshire *K. oxytoca* genomes are available on PathogenWatch. The two novel *bla*_OXY-2_ (blaOXY-2-35 and blaOXY-2-36) variants can be accessed in the BIGSdb *Klebsiella* allele database. The phylogenetic trees of global *K. oxytoca* can be viewed in iToL: Global clinicaldata set (324 genomes). Global data set from clinical + environmental + unknown sources (n = 893 genomes).

## SUPPLEMENTAL MATERIAL

Supplemental Figures S1-S7 spectrum.00549-23-s0001.pdf Supplemental Figures S1-S7

Supplemental Tables S1-S8 spectrum.00549-23-s0002.xlsx Supplemental Tables S1-S8

ASM does not own the copyrights to Supplemental Material that may be linked to, or accessed through, an article. The authors have granted ASM a non-exclusive, world-wide license to publish the Supplemental Material files. Please contact the corresponding author directly for reuse.
