# Supplementary material for: Phylogenetic lineages and antimicrobial resistance determinants of clinical Klebsiella oxytoca spanning local to global scales
Source: Microbiol Spectr. 2023 Sep 7;11(5):e00549-23. doi: 10.1128/spectrum.00549-23 (PMC10581156; doi:10.1128/spectrum.00549-23)
Supplement: Supplemental Figures S1-S7 [file spectrum.00549-23-s0001.pdf]

Phylogenetic lineages and antimicrobial resistance determinants of clinical *Klebsiella oxytoca* spanning local to global scales

Odion O. Ikhimiukor, Stephanie S. R. Souza, Michael M. Marcovici, Adrienne Workman, Isabella W. Martin, Cheryl P. Andam

Supplementary material

Supplementary Table S1. Minimum inhibitory concentration of 20 antimicrobial agents against 20 New Hampshire *K. oxytoca* isolates.

Supplementary Table S2. Accession numbers and genome sequence quality of the 20 New Hampshire *K. oxytoca* genomes and 304 clinical genomes from NCBI.

Supplementary Table S3. Accession, source, location, Multi-locus Sequence Types and collection year of the 893 *K. oxytoca* genomes from all sources retrieved from NCBI (clinical, environmental unknown sources).

Supplementary Table S4. Classification and list of genes in the pan-genome of the 20 New Hampshire *K. oxytoca* genomes sequenced in this study. The pan-genome was analyzed using Roary.

Supplementary Table S5. Associated metadata, multi-locus sequence type, antimicrobial resistance genes, virulence determinants, and plasmid replicon types detected in the 20 New Hampshire *K. oxytoca* genomes sequenced in this study.

Supplementary Table S6. Matrix showing the genome-wide average nucleotide identity (ANI) for all pairs of *K. oxytoca* genomes sequenced in this study (20 genomes from New Hampshire) and the 304 genomes retrieved from NCBI. For visual clarity, a color-coded matrix version of this table is shown in Supplementary Figure S3.

Supplementary Table S7. Classification of genes in the pan-genome of the combined 20 New Hampshire *K. oxytoca* genomes sequenced in this study and 304 globally distributed genomes retrieved from NCBI. The pan-genome was analyzed using Roary.

Supplementary Table S8. Associated metadata, multi-locus sequence type, antimicrobial resistance genes, virulence determinants, and plasmid replicon types detected in the 304 *K. oxytoca* genomes retrieved from NCBI (global clinical dataset).

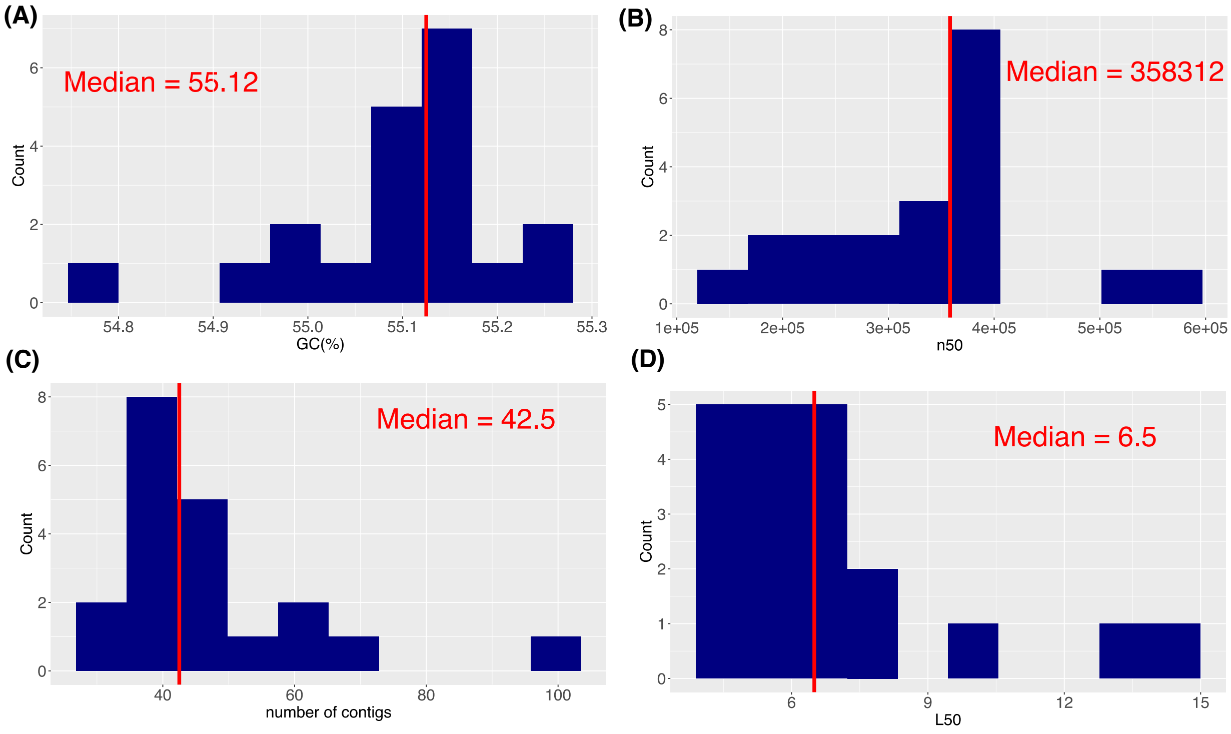

Supplementary Figure S1. Genome quality metrics (G+C percentage, N50, number of contigs, L50) of the 20 *K. oxytoca* isolates from New Hampshire sequenced in this study.

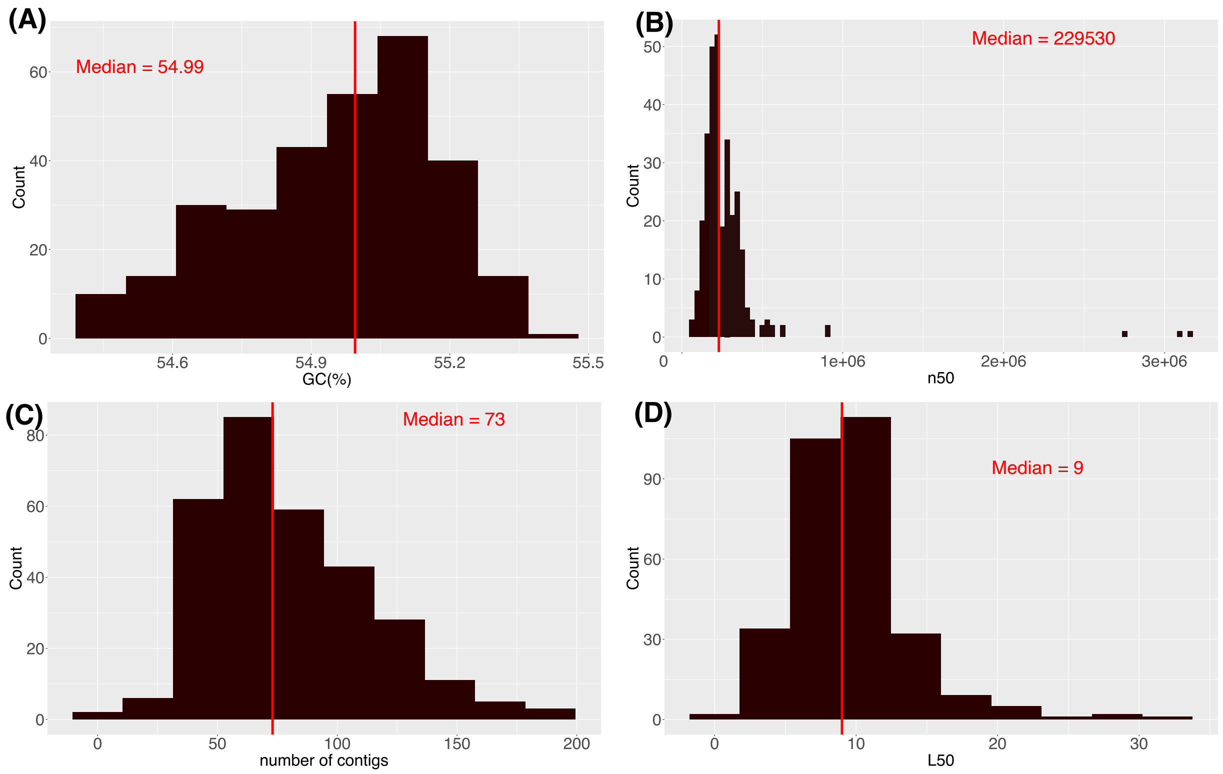

Supplementary Figure S2. Genome quality metrics (G+C percentage, N50, number of contigs, L50) of the 304 genomes from clinical *K. oxytoca* retrieved from NCBI.

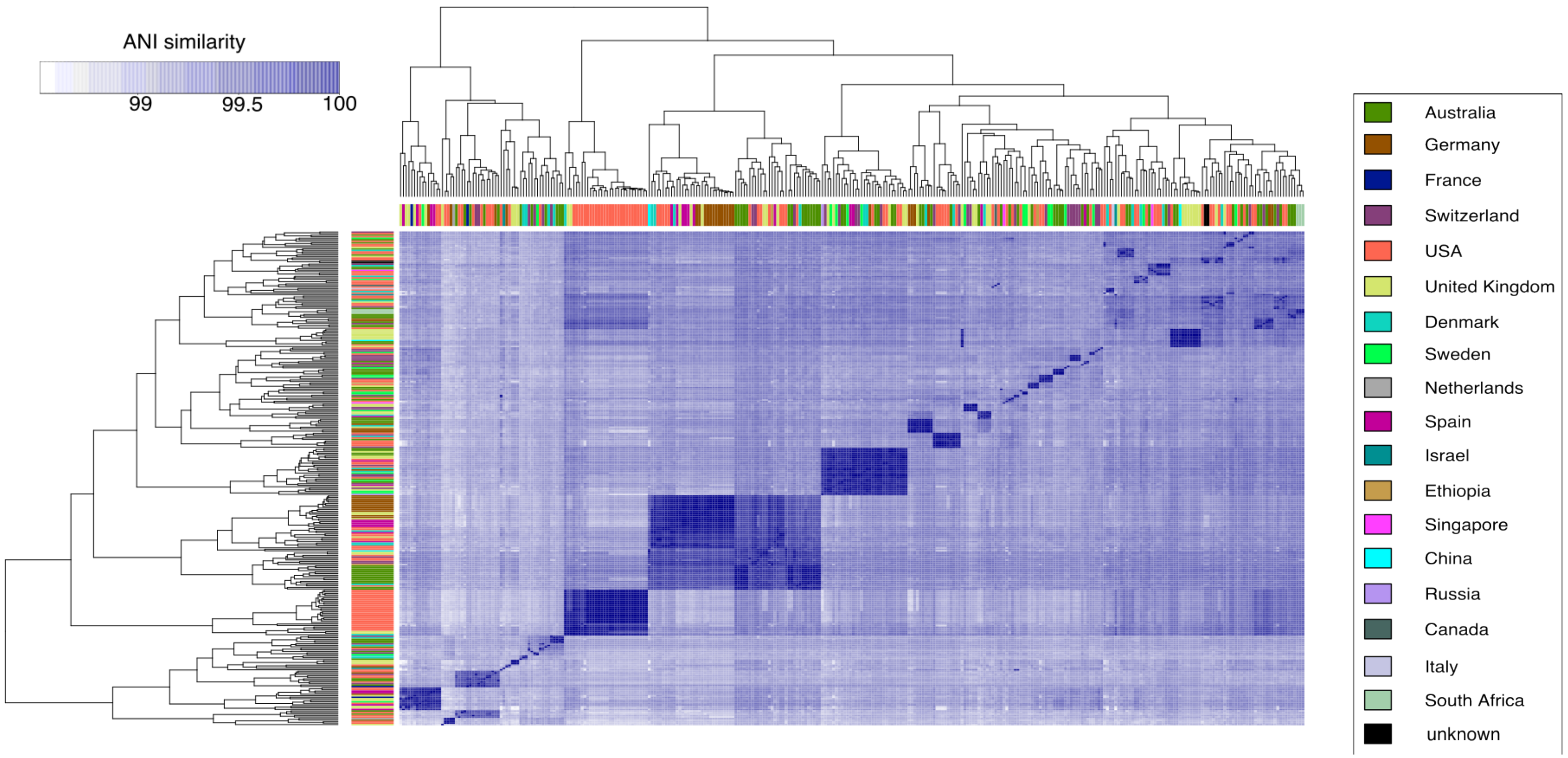

Supplementary Figure S3. Matrix showing the genome-wide average nucleotide identity (ANI) for all pairs of clinical *K. oxytoca* genomes sequenced in this study (20 genomes from New Hampshire) and the 304 genomes retrieved from NCBI. Percentage values are presented in Supplementary Table S5.

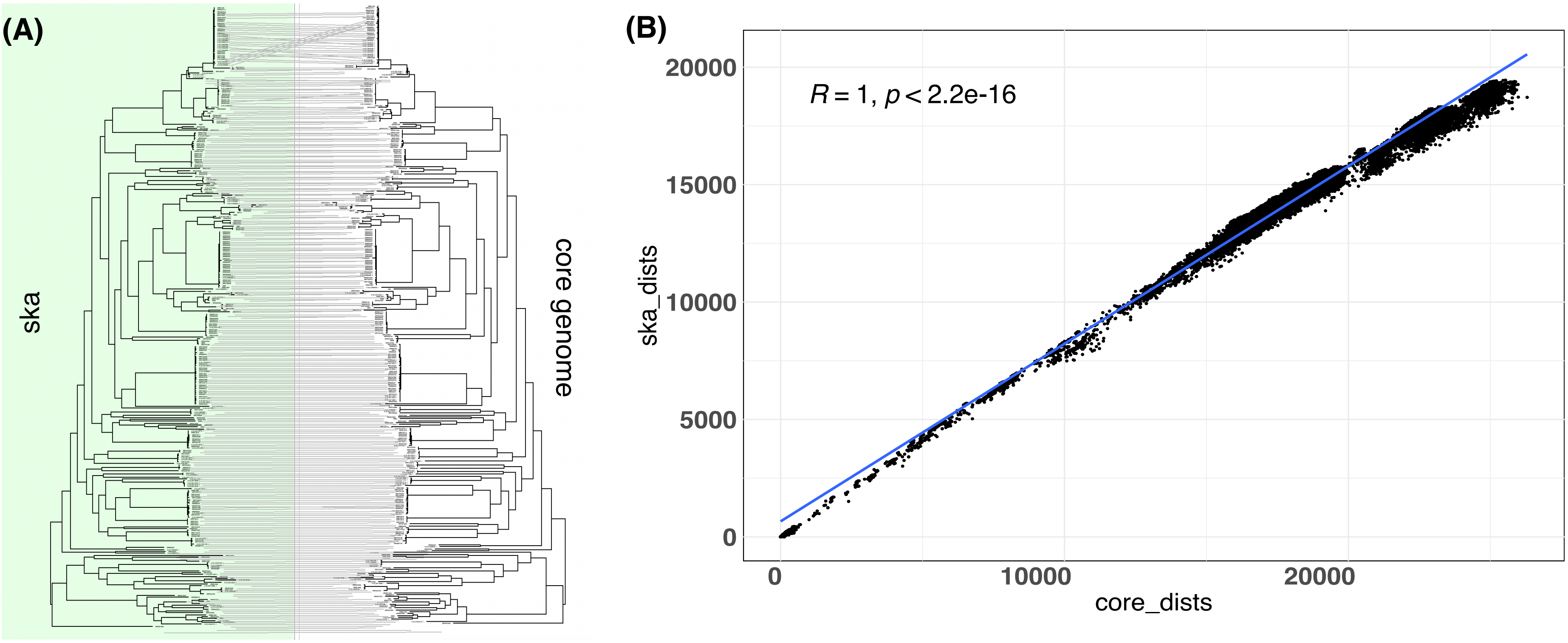

Supplementary Figure S4. Validation of Split K-mer Analysis. (A) Tanglegram of phylogenies produced by core genome and split k-mer analysis of the global clinical *Klebsiella* dataset (n = 324 genomes). (B) Correlation plots showing agreement between single nucleotide polymorphisms produced by core genome and SKA. The two variables were tested using the Pearson correlation coefficient.

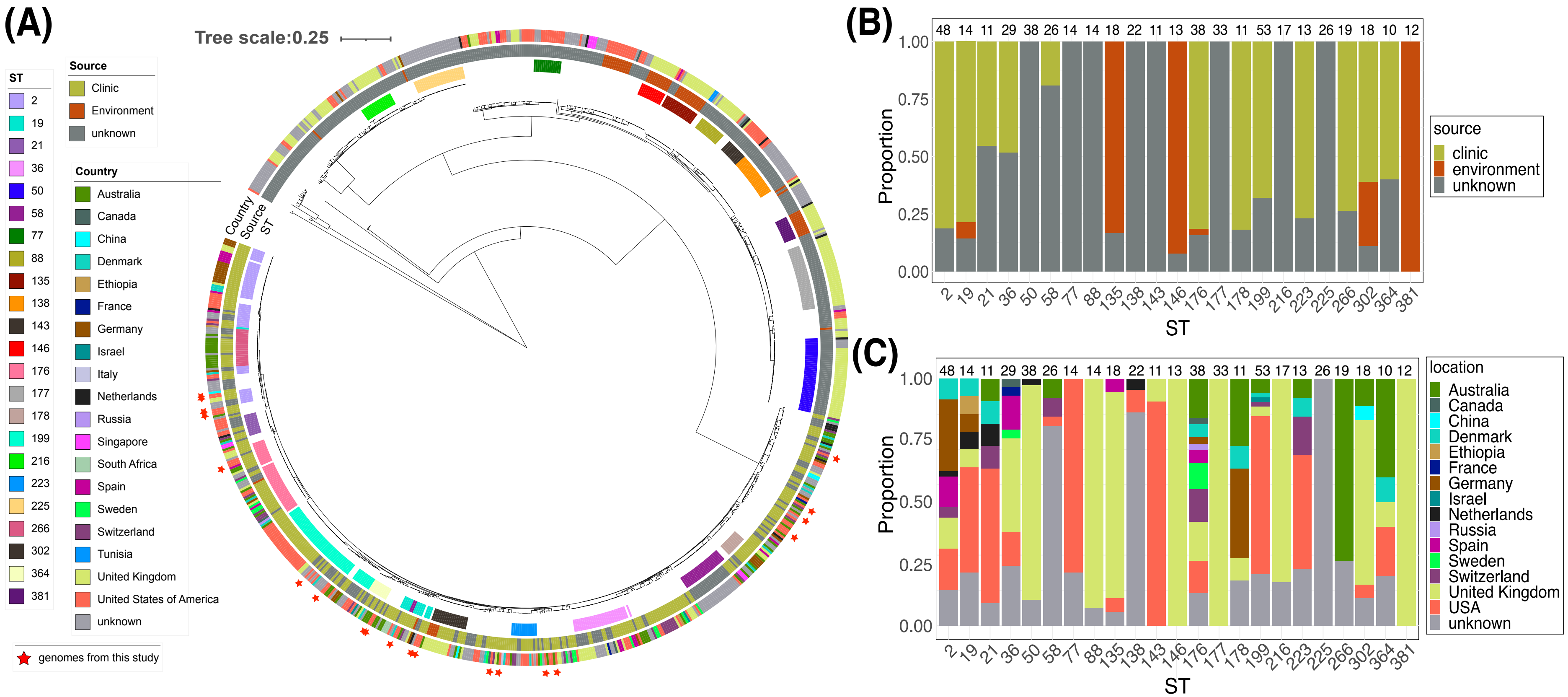

Supplementary Figure S5. Phylogenetic relationships and distribution of 893 *K. oxytoca* genomes from all sources (grouped into clinical [n = 326], environmental [n = 65], and unknown sources [n = 502]). (A) Phylogeny produced by split k-mer analysis of global *K. oxytoca* showing Sequence Types (represented by 10 or more genomes), source and country of isolation. Distribution of *K. oxytoca* Sequence Types (represented by 10 or more genomes) (B) across the different sources of isolation and (C) across the different countries of isolation.

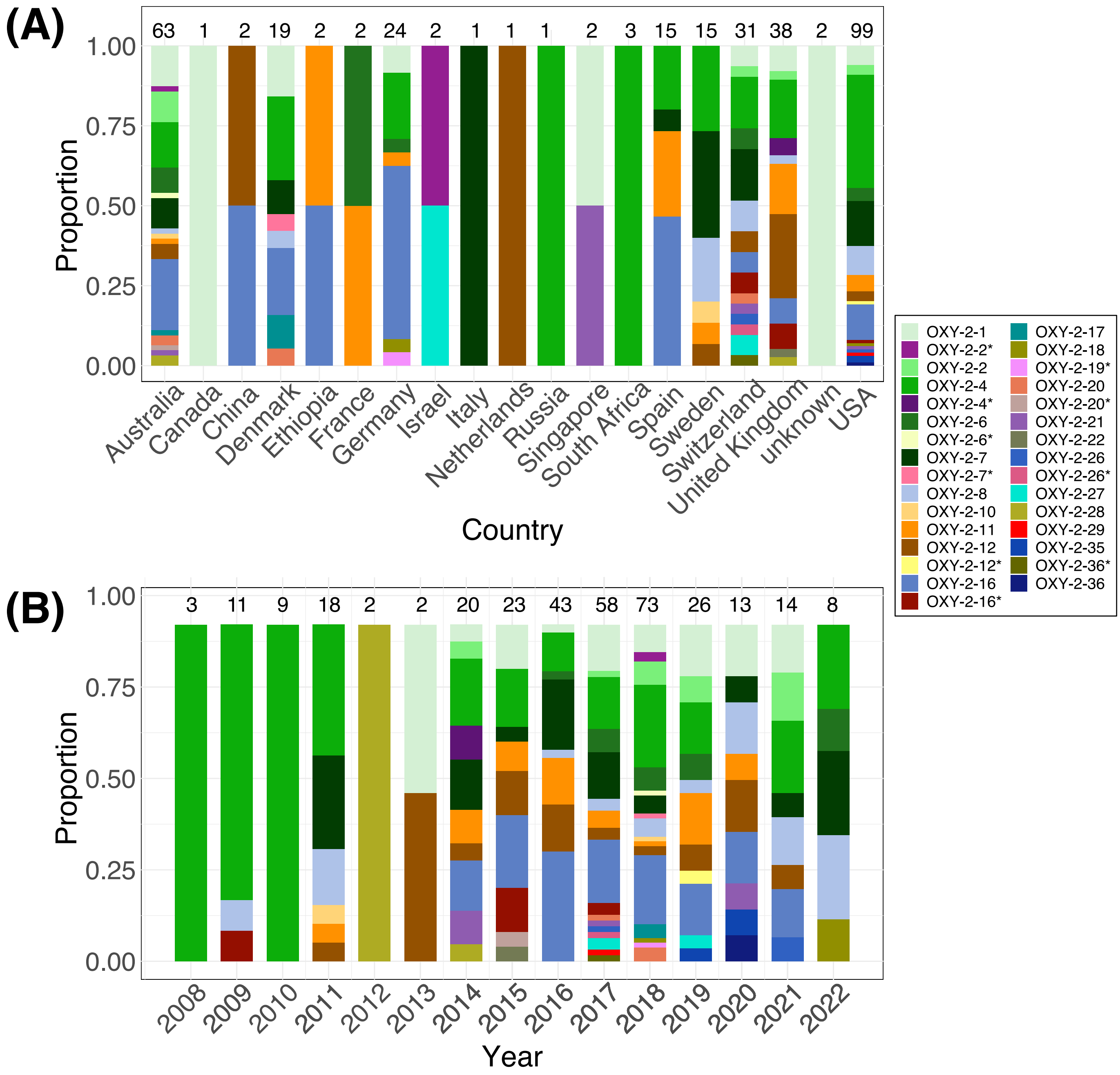

Supplementary Figure S6. Diversity and distribution of *bla*$_{\text{OXY-2}}$ in the global clinical *K. oxytoca* dataset (n = 324 genomes). Distribution and proportion of *bla*$_{\text{OXY-2}}$ variants per (A) country of origin and (B) year of isolation. For both panels, the total number of genomes per category is shown at the top of the bars. Genes not having 100% sequence similarity with *bla*$_{\text{OXY-2}}$ genes in the databases are indicated by an asterisk in the color legend.

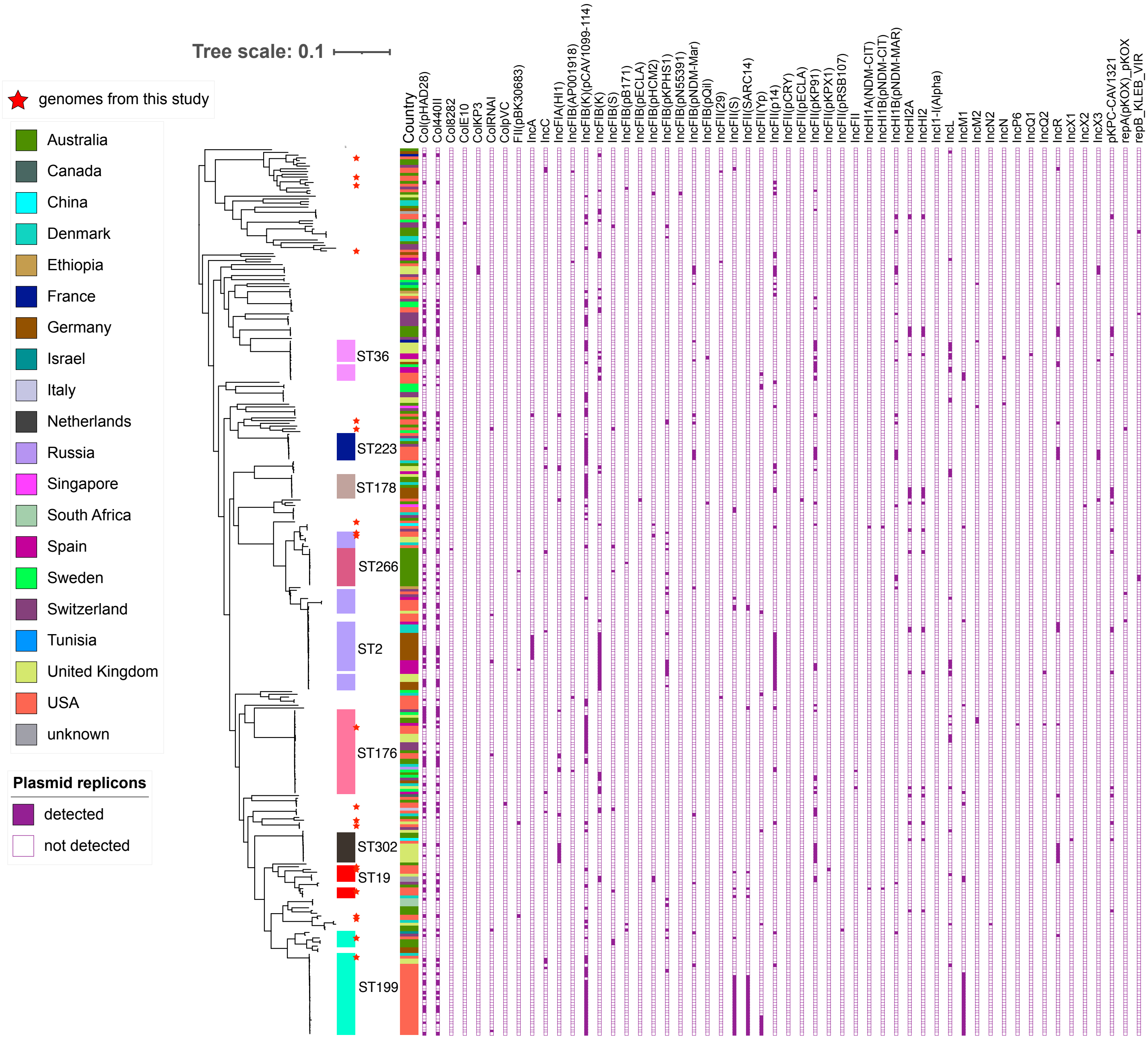

Supplementary Figure S7. Diversity and phylogenetic distribution of plasmid replicon types in the global clinical *K. oxytoca* dataset (n = 324 genomes). (A) A midpoint-rooted phylogenetic tree showing sequence types, country of isolation and a binary matrix showing the presence (purple) or absence (white) of plasmid replicons. The tree is identical to that in Figure 3A. The tree was built on the sequence alignment of 4,062 concatenated core genes. Tree scale represents the number of substitutions per site. Branches with red star is used to indicate the 20 New

Hampshire genomes sequenced in this study. For visual clarity, only the most common sequence types (STs) are labeled in color on the phylogeny.
